# Circulating tumor DNA predicts outcome in metastatic gastroesophageal cancer

**DOI:** 10.1007/s10120-022-01313-w

**Published:** 2022-06-28

**Authors:** Merel J. M. van Velzen, Aafke Creemers, Tom van den Ende, Sandor Schokker, Sarah Krausz, Roy J. Reinten, Frederike Dijk, Carel J. M. van Noesel, Hans Halfwerk, Sybren L. Meijer, Banafsche Mearadji, Sarah Derks, Maarten F. Bijlsma, Hanneke W. M. van Laarhoven

**Affiliations:** 1 Department of Medical Oncology, Cancer Center Amsterdam, Amsterdam UMC, University of Amsterdam, Meibergdreef 9, 1105 AZ Amsterdam, The Netherlands; 2 Laboratory for Experimental Oncology and Radiobiology, Center for Experimental and Molecular Medicine, Cancer Center Amsterdam, Amsterdam UMC, University of Amsterdam, Meibergdreef 9, Amsterdam, The Netherlands; 3 Department of Pathology, Amsterdam UMC, University of Amsterdam, Meibergdreef 9, Amsterdam, The Netherlands; 4 Department of Radiology, Amsterdam UMC, University of Amsterdam, Meibergdreef 9, Amsterdam, The Netherlands; 5 Department of Medical Oncology, Cancer Center Amsterdam, Amsterdam UMC, Vrije Universiteit Amsterdam, De Boelelaan 1118, Amsterdam, The Netherlands

**Keywords:** Circulating tumor DNA, Gastroesophageal cancer, Palliative treatment, Predictive factor

## Abstract

**Background:**

Circulating tumor DNA (ctDNA) has predictive and prognostic value in localized and metastatic cancer. This study analyzed the prognostic value of baseline and on-treatment ctDNA in metastatic gastroesophageal cancer (mGEC) using a region-specific next generation sequencing (NGS) panel.

**Methods:**

Cell free DNA was isolated from plasma of patients before start of first-line palliative systemic treatment and after 9 and 18 weeks. Two NGS panels were designed comprising the most frequently mutated genes and targetable mutations in GEC. Tumor-derived mutations in matched metastatic biopsies were used to validate that the sequencing panels assessed true tumor-derived variants. Tumor volumes were calculated from baseline CT scans and correlated to variant allele frequency (VAF). Survival analyses were performed using univariable and multivariable Cox-regression analyses.

**Results:**

ctDNA was detected in pretreatment plasma in 75% of 72 patients and correlated well with mutations in metastatic biopsies (86% accordance). The VAF correlated with baseline tumor volume (Pearson’s *R* 0.53, *p* < 0.0001). Detection of multiple gene mutations at baseline in plasma was associated with worse overall survival (OS, HR 2.16, 95% CI 1.10–4.28; *p* = 0.027) and progression free survival (PFS, HR 2.71, 95% CI 1.28–5.73; *p* = 0.009). OS and PFS were inferior in patients with residual detectable ctDNA after 9 weeks of treatment (OS: HR 4.95, 95% CI 1.53–16.04; *p* = 0.008; PFS: HR 4.08, 95% CI 1.31–12.75; *p* = 0.016).

**Conclusion:**

Based on our NGS panel, the number of ctDNA mutations before start of first-line chemotherapy has prognostic value. Moreover, residual ctDNA after three cycles of systemic treatment is associated with inferior survival.

**Supplementary Information:**

The online version contains supplementary material available at 10.1007/s10120-022-01313-w.

## Background

Despite the use of systemic therapy, metastatic gastroesophageal cancer (GEC) has a poor prognosis with a median overall survival of approximately eleven to fourteen months [1, 2]. The backbone of systemic treatment in the metastatic setting consists of cytotoxic agents [3], combined with checkpoint inhibitors in a selected group of patients [2, 4]. However, response to treatment varies greatly and reliable biomarkers to predict response are not available, especially for patients who are treated with chemotherapy only [5].

Circulating tumor DNA (ctDNA) is a promising prognostic and predictive factor in both localized and metastatic disease [6–9]. ctDNA, obtained through a minimally invasive liquid biopsy, could provide an easy alternative to obtain real-time information on genomic evolution and therapy response, including the induction of targetable mutations under the influence of therapeutic pressure [7, 10]. In gastroesophageal cancer, the presence of ctDNA is related to a worse prognosis and a higher disease recurrence rate after curative resection [6, 11–13].

Many studies evaluating the presence of ctDNA in GEC use panel-based next generation sequencing (NGS) platforms evaluating entire genes associated with GEC [11]. This requires an extensive bio-informatics pipeline to identify true tumor derived variants and is not easily implemented in clinical practice. The analysis of specific genomic regions frequently mutated in GEC may provide a more cost-efficient alternative to evaluate ctDNA. Importantly, the value of ctDNA in metastatic GEC has only been investigated in small cohorts, HER2 positive patients treated with trastuzumab or without extensive sampling over time [8, 14–19].

In our study we developed an NGS panel based on specific regions that are frequently mutated in GEC identified from public tumor sequencing data. We hypothesized that our panel could identify tumor-specific variants with prognostic value and inform on treatment response in patients with metastatic GEC who received first-line palliative treatment.

## Methods

### Patients and samples

We identified 72 patients from the prospective BiOES esophageal and gastric cancer biobank of the Amsterdam UMC (METC 2013_241) planned to undergo first-line treatment for metastatic gastric or esophageal cancer and for whom at least a baseline plasma sample was available. In 7 patients matched tissue samples were available for validation of the DNA detection method using the designed panels. Follow-up plasma samples were available in consecutively 28 patients after 9 weeks of treatment (first evaluation CT-scan) and 16 patients after 18 weeks of treatment (second evaluation CT-scan). Because of the small number of samples at the second evaluation CT-scan, we only used this time-point for tracking baseline mutations and for treatment response correlations, but not for survival analysis. For 19 patients only a baseline plasma sample was available. All tumor biopsies and baseline plasma samples were collected before start of chemotherapy. At the time of obtaining ctDNA samples, CT scans were available to assess treatment response.

All patients were included in the analysis of baseline ctDNA mutations; survival was only assessed in patients treated with chemotherapy (without targeted therapy) and for whom follow-up data were available (Supplementary Fig. 1).

Clinical data were extracted from the electronic patient files by a trained medical doctor and requested from other hospitals when patients were treated outside of the Amsterdam UMC.

### DNA isolation

Blood was collected in 10 mL EDTA blood collection tubes before start of first-line treatment and for patients treated at the Amsterdam UMC also after 9 weeks and 18 weeks, corresponding with the first and second response evaluation. Within one hour after collection, plasma was separated by centrifuging twice. Tubes were centrifuged for 10 min at 1.300RCF, plasma was transferred to 1.5 mL Eppendorf tubes and centrifuged again for 10 min at 20.000RCF. Plasma was stored at -80 °C until DNA isolation. Cell free DNA was isolated from 4 mL of plasma using QIAamp Circulating Nucleic Acid Kit (Qiagen, Venlo, the Netherlands) according to the manufacturer’s instructions, using a twice repeated elution. DNA extraction from matching metastatic tumor tissue was performed for seven patients using H&E slides of tumor cells. DNA was extracted via direct proteinase K digestion (Roche), according to the manufacturer’s instructions.

### Next-generation sequencing and mutation analysis

Publicly available whole exome or whole genome sequencing data from the Catalogue of Somatic Mutations in Cancer (COSMIC), cBioPortal, International Cancer Genome Consortium (ICGC) databases, and results from a literature search including whole exome/genome studies in GEC were used to identify the most prevalent mutations in GEC. The twenty most frequently mutated genes were included in our panel, along with genes identified from the literature search. The genes had to have recurrent mutations, defined as having a mutation count of at least two in COSMIC. The exonic regions of the *TP53* gene were all included because of the high prevalence of driver *TP53* mutations in GEC which are not grouped in specific hotspots [20]. The assembled genomic overview was divided into two panels: a panel with the twenty most frequently mutated genes (HF-panel) and a panel comprising the genes mutated with a low prevalence or amplified regions (LF-panel) (Supplementary Table 1). Primers were developed for mutation specific regions based on the Ampliseq pipeline (ThermoFisher, Ion Ampliseq Designer) and adjusted so that roughly equal amounts of reads were obtained for each amplicon.

DNA libraries were produced using the custom Ion AmpliSeq Panel (Life Technologies, Bleiswijk, the Netherlands) according to the manufacturer’s instructions. Libraries were barcoded with an Ion Xpress Barcodes adapters kit (Life Technologies) and quantified with a Qubit dsDNA HS assay kit (Life Technologies). The libraries were sequenced on a 318C chip on the Ion personal genome machine system (Ion Torrent, Life Technologies). The samples were sequenced to a depth of 2500 reads per amplicon on the LF panel and 5000 reads per amplicon on the HF panel.

The acquired sequencing data was analyzed in SeqNext software v.4.1.2 (JSI medical systems GmbHm, Ettenheim, Germany). Mutations were manually assessed for being true variants rather than sequencing artefacts. Variants of interest had to be located in an exonic or splice site region and should be nonsynonymous. Germline variants were removed using the ExAC, 1000 Genomes project, dbSNP and ClinVar databases. Mutations were classified as ctDNA variants if they had a deleterious or gain of function impact on the gene. This was assessed using the COSMIC, Human Gene Mutation Database (HGMD) and Ensembl archive. Mutations that were present in neither of those databases were assessed using MutationTaster, an online tool that predicts pathogenicity of mutations [21]. Mutations that were classified as polymorphisms by MutationTaster were excluded. For mutation calling of unknown ctDNA variants, a cutoff value for the variant allele fraction (VAF) was set at 1% [22]. Using this threshold we also minimized the chance of accidentally classifying clonal hematopoiesis of indeterminate potential (CHIP) variants as tumor derived. The majority of CHIP variants occur at a VAF below 1% [6, 23]. In cases with available tumor tissue sequencing data we tried to identify the tumor specific variants in the cell free DNA sequencing data in the reads even if the VAF was below 1%. Moreover, we took the same approach with the follow-up samples to track baseline mutations over time.

### Measurement of tumor volume

Baseline CT scans were used to measure three dimensional tumor volume as previously described [24]. In short semi-automated software (MM oncology, Syngo Via, Siemens Healthineers, Forchheim, Germany) was used for volume calculations (in milliliters) under the supervision of an experienced radiologist blinded for outcome. Primary lesions were included when measurable or evaluable, as well as pathologically enlarged lymph nodes and distant metastases, all according to the Response Evaluation Criteria in Solid Tumors (RECIST) 1.1. [25]

### Statistical analysis

Wilcoxon sum rank test and Pearson’s correlation coefficient were used to assess the relation between ctDNA and baseline tumor volume. For survival analyses, missing baseline variables were handled using multiple imputation with the construction of 5 databases [26]. Univariable cox regression analysis was used to investigate the association between presence and characteristics of ctDNA and overall survival (OS) or progression free survival (PFS). OS was defined as time between start of treatment and death and PFS was defined as time between start of treatment and first documented progression or death. If a patient was lost to follow-up before any of these events occurred, they were censored. Any variables that were deemed clinically significant were tested univariably and included in the multivariable analysis when they reached a *p* value below 0.1. All analyses were performed two-sided and a *p* value < 0.05 was considered statically significant. IBM SPSS Statistics for Windows version 26.0 was used for all survival analyses.

## Results

### Patient characteristics

We included 72 patients with metastatic gastric or esophageal cancer, confirmed by histology or cytology, who did not receive previous palliative treatment. Most patients were diagnosed with adenocarcinoma (83%) and in 78% the tumor was located in the esophagus. Palliative treatment consisted of capecitabine and oxaliplatin (CAPOX) in 67% of patients. Nab-paclitaxel was added to CAPOX in 22% of patients who participated in a phase II clinical trial (ACTION) [24]. Of the remaining patients, 8% received trastuzumab in addition to chemotherapy and 3% of patients did not start palliative systemic treatment. All relevant patient characteristics are listed in Table 1.

**Table 1:** Baseline characteristics

	All patients (*n* = 72)	Patients included in survival analyses (*n* = 63)
Age—median (IQR)	65 (60–69)	64 (59–68)
Sex—male (%)	56 (77.8)	47 (74.6)
Performance status		
0–1	65 (90.2)	56 (91.8)
≥ 2	5 (6.9)	5 (8.2)
Missing	2 (2.8)	0
Location of primary tumor		
Esophagus	56 (77.8)	48 (76.2)
Stomach	4 (5.6)	4 (6.3)
Gastroesophageal junction	12 (16.7)	11 (17.5)
Histology		
Adenocarcinoma	60 (83.3)	51 (81.0)
SCC	11 (15.3)	11 (17.5)
Other	1 (1.3)	1 (1.6)
Differentiation grade		
Poorly differentiated	28 (38.9)	26 (41.3)
Moderately differentiated	27 (37.5)	24 (38.1)
Well differentiated	1 (1.4)	1 (1.6)
Missing	16 (22.3)	12 (19.0)
Number of metastatic sites		
1	27 (37.5)	23 (36.5)
2	27 (37.5)	24 (38.1)
3 or more	18 (25.0)	16 (25.4)
Albumin—median (IQR)	41 (37–45)	39 (37–41)
LDH—median (IQR)	220 (177–347)	198 (171–249)
Previous chemo(radio)therapy		
Yes	34 (47.2)	31 (49.2)
No	37 (51.4)	31 (49.2)
Unknown	1 (1.4)	1 (1.6)
Previous surgery		
Yes	19 (26.4)	45 (71.4)
No	52 (72.2)	17 (27.0)
Missing	1 (1.4)	1 (1.6)
Palliative treatment		
CapOx	48 (66.7)	47 (74.6)
CapOx-nab-P	16 (22.2)	16 (25.4)
CapOx-T^a^	6 (8.3)	0 (0.0)
Supportive care	2 (2.7)	0 (0.0)
Second-line palliative treatment		
Yes	24 (33.3)	23 (36.5)
No	38 (52.8)	34 (54.0)
Missing	10 (13.9)	6 (9.5)
Tumor volume—median cm^3^ (IQR)	78.5 (38.2–124.1)	
ctDNA detected		
Yes	53 (75.0)	47 (74.6)
No	18 (25.0)	16 (25.4)
ctDNA MAF—median (IQR)^a^	5% (2–13%)	5% (1–12%)

^a^One patient was treated with CapOx, trastuzumab and pertuzumab in the JACOB trial

### Accordance between ctDNA, tissue DNA and tumor volume

We first evaluated if our ctDNA panel could detect tumor specific variants by sequencing both cell free DNA and tumor tissue and comparing mutations in SeqNext. In six out of seven plasma samples used for baseline comparison, ctDNA was detected and agreement between tissue and ctDNA mutations was high (86%; Table 2). One mutation found in the tumor biopsy was not identified in the baseline plasma sample (patient 034) and three mutations found in the baseline plasma sample were not present in the tumor biopsies (patients 034, 005 and 039).

**Table 2:** Accordance of mutations in tumor biopsies and plasma ctDNA by VAF

Patient	Mutations	Tissue		Blood	
		Baseline	Baseline	Follow-up 9 weeks	Follow-up 18 weeks
005	TP53 c.524G>A	28%	2%	< 1%	6%^a^
	FBXW7 c.1177dupA	–	9%	< 1%	–
013	TP53 c.743G > A	44%	13%	< 1%	< 1%
023	KRAS c.35G > A	–	–	–	11%
034	TP53 c.380C > T	11%	–	–	–
	KRAS c.35G > A	–	2%	–	–
039	TP53 c.743G > A	4.65%	5%	< 1%	< 1%
	CDKN2A c.35C > T	–	1%	< 1%	–
040	TP53 c.839G > A	2.40%	12%	–	1%^a^
	TP53 c.844C > T	19%	< 1%	–	–
042	TP53 c.916C > T	83%	13%	< 1%	2%

^a^Samples drawn at time of progression on CT evaluation

**Fig. 1:**
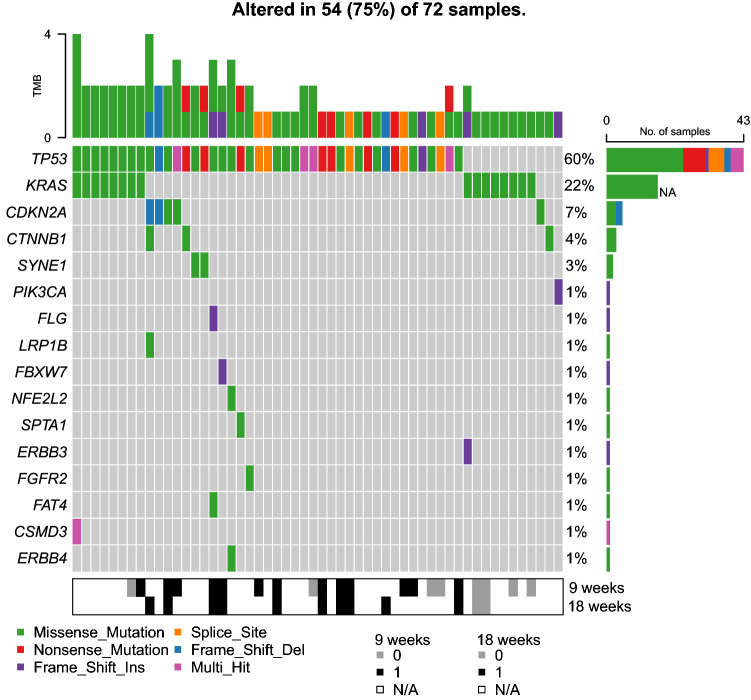
Oncoplot of baseline mutations detected in plasma ctDNA. All mutations detected after 9 or 18 weeks of treatment were also present in the baseline plasma of the same patient, except for one *TP53* mutation in exon 8 and one *KRAS* mutation in exon 2

**Fig. 2:**
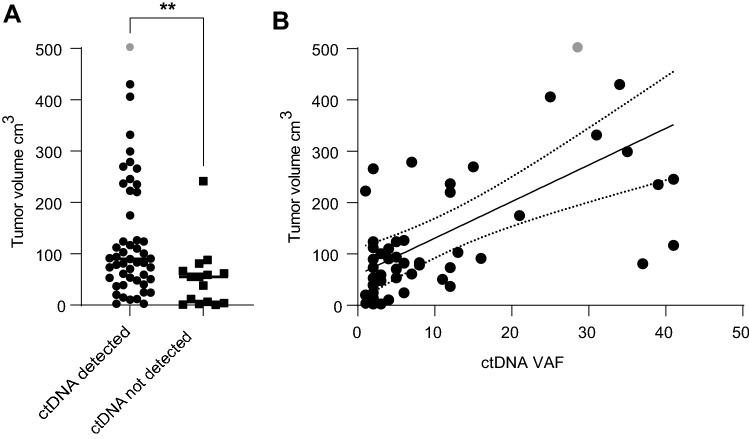
Correlation between ctDNA and Tumorvolume at baseline. **a** The median tumor volumes were compared between patients in whom ctDNA was detected versus not detected (p<0.01) **b** The correlation of ctDNA VAF in baseline samples and tumor volume measured in cm^3^ by 3D imaging was assessed using lineair correlation analysis. Pearson’s correlation coefficient 0.53, *p* < 0.0001. The association between tumor volume in cm^3^ and variant allele frequency (VAF) in percentages was analysed using simple linear regression. *R*-squared 0.28, *p* < 0.0001. The grey dot represents a patient with a VAF of 28% and a tumor volume of 982 cm^3^

In 75% of 72 baseline plasma samples ctDNA was detected with a median variant allele frequency of 5%. Mutations most frequently occurred in *TP53* (60%) and *KRAS* (22%). TP53 mutations could be found on several locations and were not bound to one specific hotspot. The most prominent hotspot KRAS mutation was c.35G > A in 81% (*n* = 13) of patients with a KRAS mutation. A mutation in two or more genes was present in 21 of 72 patients (29%; Fig. 1). We observed a strong relationship between the presence of ctDNA and baseline tumor volume. Patients with detectable ctDNA had a higher median tumor volume (median 90.1 cm^3^, IQR 50.1–220.9) compared to patients who were ctDNA negative (median 55.2 cm^3^, IQR 3.8–66.4; Fig. 2a). Moreover, the maximum VAF in patients with detectable ctDNA was strongly correlated with tumor volume (Pearson’s R = 0.5266, *p* < 0.0001; Fig. 2b).

### Prognostic value of baseline ctDNA and treatment response

**Fig. 3:**
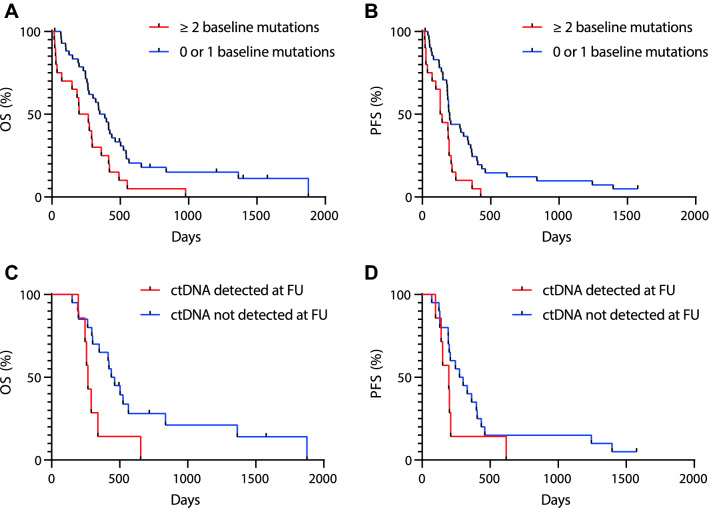
Association between ctDNA and outcome. Overall survival (**a**) and progression free survival (**b**) for patients separated by number of baseline mutations, analyzed by Kaplan–Meier and cox regression. Hazard ratio for OS was 2.01 (95% CI 1.15–3.50; *p* = 0.014) and for PFS 2.12 (95%CI 1.20–3.74, *p* = 0.009). Overall survival (**c**) and progression free survival (**d**) for patients separated by presence of ctDNA with a VAF of 1% or more in the follow up (FU) plasma after 9 weeks of treatment. Hazard ratio for OS was 2.74 (95%CI 1.07–6.98, *p* = 0.035z) and for PFS 1.87 (95%CI 0.76–4.59, *p* = 0.171)

For survival analysis, we compared patients with no or only one detectable mutation with patients with two or more detectable mutations. Patients who had two or more detectable mutations showed worse OS (HR 2.01, 95% CI 1.15–3.50; p-value 0.014) and PFS (HR 2.12, 95% CI 1.20–3.74; p-value 0.009) compared to patients with only one or no detectable mutations in univariate analysis (Fig. 3a, b). Moreover, in multivariable analysis, patients with two or more ctDNA variants also showed worse OS (HR 2.16, 95% CI 1.10–4.28, *p* = 0.027) and PFS (HR 2.71, 95% CI 1.28–5.73, *p* = 0.009) compared to patients with no mutations or only a single mutation (Table 3 and Supplementary Table 2 and 3). As ctDNA and tumor volume were correlated, we assessed if tumor volume could also predict survival. There was however no difference between patients with a high tumor volume (> median) or low tumor volume (< median), OS (HR 0.93, 95% CI 0.53–1.61; *p* = 0.79) and PFS (HR 0.90, 95% CI 0.53–1.53; *p* = 0.69).

**Table 3:** Uni- and multivariable regression analyses for OS and PFS (patients treated with trastuzumab were excluded) according to number of mutations at baseline

	*N*	Univariable analysis			Multivariable analysis^a^		
		HR	95% CI	*p* value	HR	95% CI	*p* value
Overall survival							
Number of mutations							
0 or 1	43	Ref			Ref		
≥ 2	20	2.008	1.152–3.499	0.014	2.164	1.095–4.276	0.027
Progression free survival							
Number of mutations							
0 or 1	43	Ref			Ref		
≥ 2	20	2.120	1.203–3.736	0.009	2.710	1.282–5.726	0.009

^a^Multivariable Cox regression analysis for overall survival was adjusted for age, WHO performance score, primary tumor site and previous surgery. Multivariable analysis for progression free survival was adjusted for age, WHO performance score, primary tumor site, previous surgery and previous chemoradiotherapy. See also Supplementary Table 2

Subsequently, we investigated if residual ctDNA after 9 weeks of treatment is associated with response and survival. Indeed, patients with detectable ctDNA (1 or more mutations with a VAF > 1%) after 9 weeks of treatment had worse OS (HR 4.95, 95% CI 1.53–16.04; *p* = 0.008) and PFS (HR 4.08, 95% CI 1.31–12.75; *p* = 0.016) in a multivariable regression analysis compared to patients with no detectable ctDNA in their first follow-up sample (Fig. 3c, d; Table 4 and Supplementary Table 4 and 5). In patients who had a follow-up sample available after 18 weeks of treatment we also observed a relationship between VAF and response to treatment, however this association was not statistically significant (Supplementary Fig. 2, *p* = 0.07).

**Table 4:** Uni- and multivariable regression analyses for OS and PFS (patients treated with trastuzumab were excluded) according to residual ctDNA at follow-up after 9 weeks of treatment

*N*		Univariable analysis			Multivariable analysis^a^		
		HR	95% CI	*p* value	HR	95% CI	*p* value
Overall survival							
Residual detectable ctDNA at follow-up							
< 1%	20	Ref			Ref		
≥ 1%	7	2.736	1.073–6.977	0.035	4.946	1.525–16.040	0.008
Progression free survival							
Residual detectable ctDNA at follow-up							
< 1%	20	Ref			Ref		
≥ 1%	7	1.87	0.76–4.59	0.171	4.08	1.31–12.75	0.016

^a^Multivariable Cox regression analyses for overall survival and progression free survival were adjusted for age and primary tumor site. See also Supplementary Table 3

## Discussion

With our newly developed targeted NGS panel, based on frequently mutated regions in GEC, we were able to detect ctDNA in a cohort of 72 metastatic GEC patients. We have established the prognostic value of ctDNA and its relation to treatment response. To our knowledge, this is the biggest patient cohort to find an association between the number of baseline mutations and survival in metastatic GEC treated with first-line chemotherapy. We also observed an association between residual ctDNA after three cycles of chemotherapy and survival, corresponding with other data published regarding ctDNA in metastatic gastroesophageal cancer [7, 9].

We observed worse survival in patients treated with first-line chemotherapy with more than one mutation in their baseline plasma. This is in line with baseline ctDNA findings from HER2 + positive trastuzumab treated GEC cohorts [17, 19]. In a cohort of 31 metastatic gastric cancer patients the molecular tumor burden index (mean VAF of clonal mutations) was predictive of PFS [17]. In the INTEGA trial, investigating different first line immunotherapy regimens for HER2 + GEC, ctDNA load was also associated with shorter PFS/OS [19]. It thus seems several ctDNA metrics at baseline are prognostic and might indicate more aggressive cell turnover as the shedding of ctDNA is related to apoptosis and mitosis of cancer cells [27]. Interestingly, in this study we showed that patients with a higher tumor volume present with higher levels of ctDNA, while tumor volume itself was not associated with survival. This suggests that the presence of ctDNA not only reflects the total volume of tumor in the body, but other factors intrinsic to the tumor as well, e.g. aggressiveness. *TP53* was found to be the most commonly mutated gene, in line with previous reports on *TP53* mutations detected in up to two-thirds of metastatic GEC patients [28].

In line with recent literature, we were able to show that residual ctDNA during treatment is associated with response and can be used as an on-treatment predictive biomarker for duration of response. Due to changes in tumor characteristics during chemotherapy and targeted therapy, treatment resistant subclones may evolve and drive progression in patients [10]. The recent PANGEA trial revealed superior treatment outcomes when post-treatment biopsies guided the next-line treatment choices [29]. Despite the advantage of individualized treatment based on longitudinal investigation of tumor mutations, disadvantages of repeated biopsies should not be ignored [30, 31]. It is possible that in the near future ctDNA from liquid biopsies will be able to guide next-line treatment choices without the need for repeated biopsies. Our NGS mutation panel can easily be adjusted to incorporate new targetable mutations in GEC.

An important limitation of our study is that it remains uncertain whether patients without detectable ctDNA have a tumor that does not shed any or only very low amounts of ctDNA, or that the ctDNA is present, but not detected by our method. To improve this limitation, we need to improve the sensitivity of our sequencing assay by increasing the sequencing depth and the amount of ctDNA. Eventually we hope to improve the prognostic value of the panel and the capacity to detect targetable mutations could lead to personalized treatment options.

Another limitation of our study is that we did not sequence DNA extracted from leukocytes to account for clonal hematopoiesis of indeterminate potential variants, which can mainly be found in *TP53* and *KRAS.* These may generate false positive results. However, CHIP variants occur mainly below a VAF of 1% and we put our threshold at 1% to minimize the influence of CHIP [6, 17]. Moreover, we extensively screened public databases to account for non-pathogenic variants and only included mutations which had an effect on protein function. We also observed a strong correlation between tumor volume and VAF of tumor mutations, suggesting the variants we found to be true tumor derived mutations. This was also confirmed by our cohort of tumor tissue and ctDNA matched patients. Thus, although we cannot completely rule out the presence of CHIP variants in our sequencing results for *TP53*, the influence of these variants will probably be minimal in our cohort.

The analyses in this study were performed retrospectively and we were unable to retrieve all baseline and follow-up data from all patients. Despite our efforts to adjust for missing data, optimal adjustment for all baseline values such as previous treatment was not possible and one patient with incomplete follow-up data was therefore excluded from the survival analyses.

Furthermore, we only collected plasma samples after 9 and 18 weeks of treatment. We did not draw plasma samples from earlier time-points. Future studies should investigate samples drawn earlier in their treatment regimen to assess if early drug rotation based on ctDNA dynamics is feasible.

## Conclusions

We developed a region-specific NGS sequencing panel able to detect tumor-derived mutations from metastatic GEC patients in plasma. We established the prognostic value of ctDNA at baseline in patients who were treated with first-line chemotherapy. Moreover, the presence of ctDNA during systemic treatment was related to lack of response and worse survival. Future studies should aim to develop even more sensitive methods of ctDNA detection (e.g. by indexing individual template molecules with a unique identifier before PCR and sequencing) to find targetable mutations and look into the dynamics of ctDNA during disease progression.

## Supplementary Information

Below is the link to the electronic supplementary material.

Supplementary file1 Supplementary Figure 1. Flowchart of patient inclusion and available samples. (PDF 215 KB)

Supplementary file2 Supplementary Figure 2. ctDNA correlated with response. Correlation between objective response (OR, defined as partial or complete response according to RECIST 1.1 criteria) after 18 weeks of treatment and variant allele frequency (VAF) in percentages of ctDNA in corresponding follow up samples. (PDF 98 KB)

Supplementary file3 (DOCX 36 KB)

## Abbreviations

GEC: Gastroesophageal cancer
OS: Overall survival
ctDNA: Circulating tumor Deoxyribonucleic acid
NGS: Next generation sequencing
CT: Computed tomography
COSMIC: Catalogue of somatic mutations in cancer
ICGC: International Cancer Genome Consortium
HF panel: High frequent panel
LF panel: Low frequent panel
HGMD: Human genome mutation database
VAF: Variant allele frequency
CHIP: Clonal hematopoiesis of indeterminate potential
RECIST: Response evaluation criteria in solid tumors
PFS: Progression free survival
CAPOX: Capecitabine and oxaliplatin
IQR: Interquartile range
