# Supplementary figures and images for: Circulating tumor DNA predicts outcome in metastatic gastroesophageal cancer
Source: Gastric Cancer. 2022 Jun 28;25(5):906–15. doi: 10.1007/s10120-022-01313-w (PMC9365750; doi:10.1007/s10120-022-01313-w)

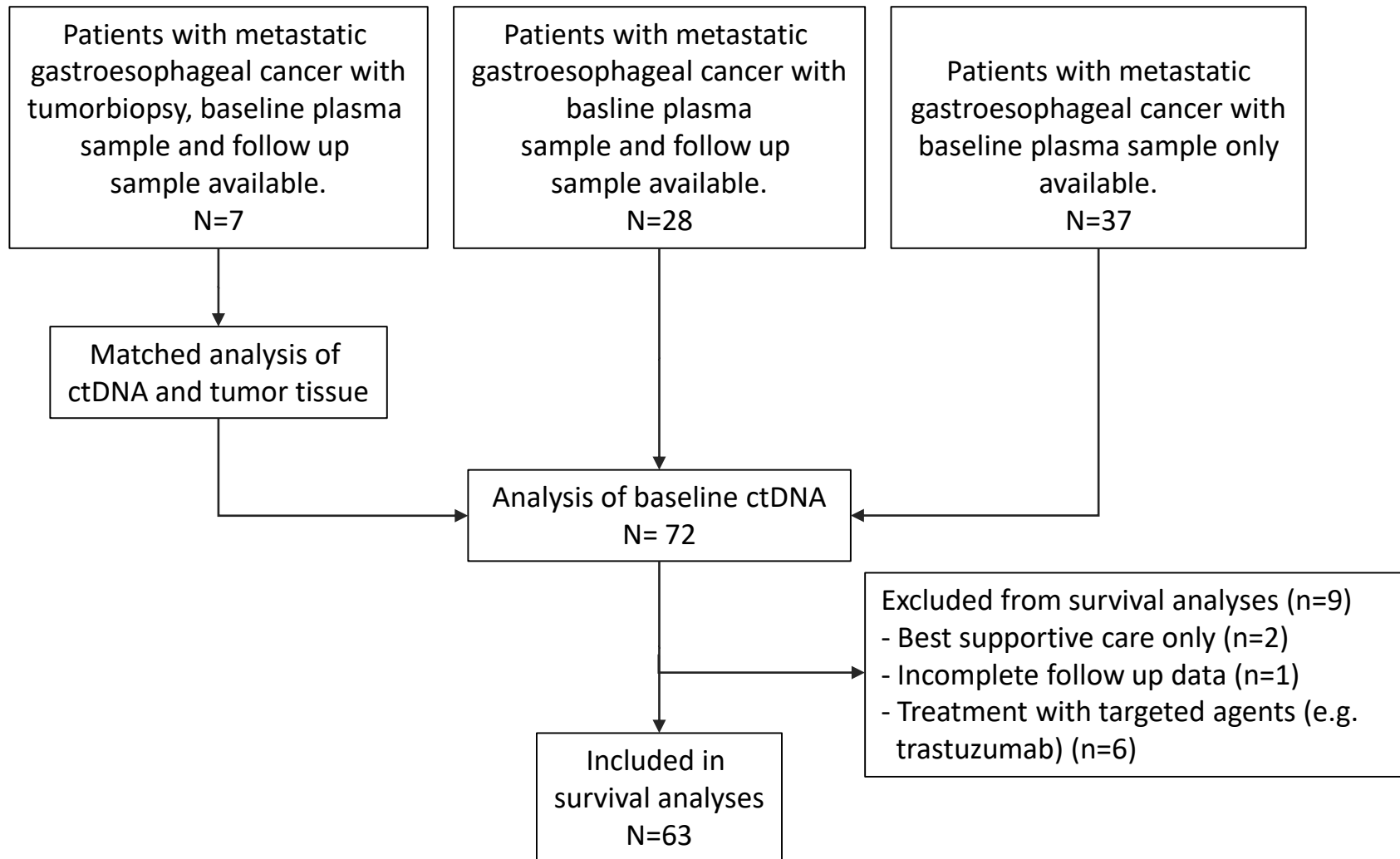

Supplement: Supplementary file 1 — Supplementary file1 Supplementary Figure 1. Flowchart of patient inclusion and available samples. (PDF 215 KB) [file 10120_2022_1313_MOESM1_ESM.pdf]

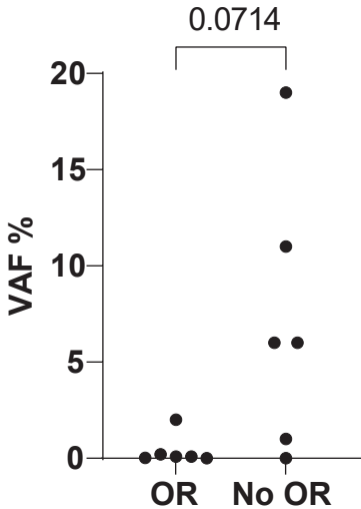

Supplement: Supplementary file 2 — Supplementary file2 Supplementary Figure 2. ctDNA correlated with response. Correlation between objective response (OR, defined as partial or complete response according to RECIST 1.1 criteria) after 18 weeks of treatment and variant allele frequency (VAF) in percentages of ctDNA in corresponding follow up samples. (PDF 98 KB) [file 10120_2022_1313_MOESM2_ESM.pdf]
