# Supplementary material for: Circulating tumor DNA predicts outcome in metastatic gastroesophageal cancer
Source: Gastric Cancer. 2022 Jun 28;25(5):906–15. doi: 10.1007/s10120-022-01313-w (PMC9365750; doi:10.1007/s10120-022-01313-w)
Supplement: Supplementary file 3 — Supplementary file3 (DOCX 36 KB) [file 10120_2022_1313_MOESM3_ESM.docx]

**Supplementary Table 1**. Mutations included in the high frequent and low frequent sequencing panel.

| Genes included in the HF panel - | *ARID1A, CNTNAP5, CTNNB1, KRAS, LRP1B, PCLO, PIK3CA, PNILPRP3, SYNE1, CSMD3, FLG, SMAD4, SPTA1, TP53, CDKN2A* |
| --- | --- |
| Genes included in the LF panel | *ABCA13, AJAP1, AKAP6, ALK, APC, APOB, ARID1A, CACNA1E, CNTN6, CNTNAP5, CSMD1, CSMD3, CTNNB1, EGFR, ELMO1, ERBB2, ERBB3, EYS, FAT1, FAT3, FAT4, FBXW7, FLG, GPR98/ADGRV1, HECW1, KCNQ3, KRAS, MUC16, NALCN, NOTCH1, NOTCH2, NOTCH3, NRG1, PCDH9, PIK3CA, PNILPRP3, RB1, RIMS2, RYR2, RYR3, SCN10A, SMAD4A, SMARCA4, SPG20, SPTA1, TLL1, TLR4, TRIM58, TTN* |

**Supplementary Table 2**. Uni- and multivariable regression analyses for OS (patients treated with trastuzumab were excluded) according to number of mutations at baseline.

|  | N | **Univariable analysis** | | | **Multivariable analysis** | | |
| --- | --- | --- | --- | --- | --- | --- | --- |
|  |  | HR | 95% CI | P | HR | 95% CI | P |
| **Number of mutations**  0 or 1  ≥2 | 43  20 | **Ref**  **2.008** | **1.152-3.499** | **0.014** | *Ref*  2.164 | 1.095-4.276 | 0.027 |
| **Age at diagnosis** | - | 0.979 | 0.948-1.012 | 0.212 | 0.930 | 0.895-0.967 | 0.000 |
| **WHO performance score**  WHO PS 0  WHO PS 1  WHO PS 2  Missing | 40  16  5  2 | ***Ref***  **0.827**  **2.933** | **0.442-1.545**  **1.099-7.827** | **0.551**  **0.032** | *Ref*  0.552  3.673 | 0.264-1.154  1.288-10.476 | 0.114  0.015 |
| **Albumin** | - | 0.925 | 0.808-1.058 | 0.226 | - |  |  |
| **LDH** | - | 1.000 | 0.998-1.002 | 0.984 | - |  |  |
| **Primary tumor site**  Esophagus  Stomach  GE Junction | 48  4  11 | ***Ref***  **0.896**  **0.413** | **0.320-2.507**  **0.190-0.897** | **0.835**  **0.026** | *Ref*  1.335  0.490 | 0.446-3.991  0.206-1.164 | 0.605  0.146 |
| **Number of metastatic** **sites**  1  2  ≥3 | 23  24  16 | *Ref*  0.893  0.795 | 0.490-1.626  0.400-1.580 | 0.710  0.513 | - |  |  |
| **First-line treatment**  CapOx  CapOx-nabPaclitaxel | 47  16 | *Ref*  0.863 | 0.469-1.591 | 0.637 | - |  |  |
| **Previous surgery**  No  Yes  Missing | 45  17  1 | ***Ref***  **1.667** | **0.935-2.974** | **0.083** | *Ref*  1.958 | 1.031-3.719 | 0.040 |
| **Previous chemo(radio)therapy**  No  Yes  Missing | 31  31  1 | *Ref*  1.457 | 0.853-2.488 | 0.169 |  |  |  |
| **Subsequent therapy**  No  Yes  Missing | 34  23  6 | *Ref*  0.672 | 0.380-1.188 | 0.171 |  |  |  |

Variables in bold are used for adjustment in the multivariable analysis.

**Supplementary Table 3**. Uni- and multivariable regression analyses for PFS (patients treated with trastuzumab were excluded) according to number of mutations at baseline.

|  | N | **Univariable analysis** | | | **Multivariable analysis** | | |
| --- | --- | --- | --- | --- | --- | --- | --- |
|  |  | HR | 95% CI | P | HR | 95% CI | P |
| Number of mutations  0 or 1  ≥2 | 43  20 | **Ref**  **2.120** | **1.203-3.736** | **0.009** | *Ref*  2.710 | 1.282-5.726 | 0.009 |
| **Age at diagnosis** | - | **0.961** | **0.930-0.994** | **0.019** | 0.930 | 0.895-0.967 | 0.000 |
| **WHO performance score**  WHO PS 0  WHO PS 1  WHO PS 2  Missing | 40  16  5  2 | ***Ref***  **0.946**  **4.895** | **0.511-1.439**  **1.474-16.257** | **0.860**  **0.010** | *Ref*  0.520  5.487 | 0.242-1.121  1.552-19.404 | 0.095  0.009 |
| **Albumin** | - | 0.911 | 0.812-1.022 | 0.107 | - |  |  |
| **LDH** | - | 1.001 | 0.999-1.002 | 0.368 | - |  |  |
| **Primary tumor site**  Esophagus  Stomach  GE Junction | 48  4  11 | ***Ref***  **0.679**  **0.449** | **0.243-1.901**  **0.220-0.913** | **0.462**  **0.027** | *Ref*  1.345  0.540 | 0.437-4.136  0.235-1.241 | 0.606  0.146 |
| **Number of metastatic** **sites**  1  2  ≥3 | 23  24  16 | *Ref*  0.871  0.986 | 0.475-1.595  0.514-1.893 | 0.654  0.967 | - |  |  |
| **First-line treatment**  CapOx  CapOx-nabPaclitaxel | 47  16 | *Ref*  0.807 | 0.450-1.447 | 0.471 | - |  |  |
| **Previous surgery**  No  Yes  Missing | 45  17  1 | ***Ref***  **1.870** | **1.044-3.350** | **0.035** | *Ref*  2.011 | 0.786-3.712 | 0.176 |
| **Previous chemo(radio)therapy**  No  Yes  Missing | 31  31  1 | ***Ref***  **1.657** | **0.982-2.795** | **0.059** | *Ref*  1.708 | 0.637-3.650 | 0.343 |

Variables in bold are used for adjustment in the multivariable analysis.

**Supplementary Table 4.** Uni- and multivariable regression analyses for OS (patients treated with trastuzumab were excluded) according to residual ctDNA at follow up after 9 weeks of treatment.

|  | N | **Univariable analysis** | | | **Multivariable analysis** | | |
| --- | --- | --- | --- | --- | --- | --- | --- |
|  |  | HR | 95% CI | P | HR | 95% CI | P |
| **Residual detectable ctDNA at follow up**  <1%  ≥1% | 20  7 | ***Ref***  **2.736** | **1.073-6.977** | **0.035** | Ref  4.946 | 1.525-16.040 | 0.008 |
| **Age at diagnosis** |  | **0.960** | **0.919-1.004** | **0.074** | 0.938 | 0.885-0.994 | 0.029 |
| **WHO performance score**  WHO PS 0  WHO PS 1  WHO PS 2 | 20  6  1 | *Ref*  *1.320*  *5.019* | 0.513-3.394  0.577-43.693 | 0.565  0.144 |  |  |  |
| **Albumin** |  | 0.926 | 0.777-1.104 | 0.393 |  |  |  |
| **LDH** |  | 1.003 | 0.997-1.009 | 0.331 |  |  |  |
| **Primary tumor site**  Esophagus  Stomach  GE Junction | 22  1  4 | ***Ref***  **0.386**  **0.143** | **0.049-3.037**  **0.031-0.656** | **0.365**  **0.012** | Ref  0.097  0.114 | 0.009-1.058  0.022-0.595 | 0.056  0.010 |
| **Number of metastatic** **sites**  1  2  ≥3 | 10  10  7 | Ref  0.926  0.713 | 0.363-2.367  0.242-2.099 | 0.873  0.539 |  |  |  |
| **First-line treatment**  CapOx  CapOx-nabPaclitaxel | 12  15 | Ref  1.388 | 0.588-3.277 | 0.455 |  |  |  |
| **Previous surgery**  No  Yes | 21  6 | Ref  1.685 | 0.655-4.335 | 0.279 |  |  |  |
| **Previous chemo(radio)therapy**  No  Yes | 13  14 | Ref  1.614 | 0.704-3.698 | 0.258 |  |  |  |
| **Subsequent therapy**  No  Yes  Missing | 12  13  2 | Ref  1.150 | 0.469-2.823 | 0.760 |  |  |  |

Variables in bold are used for adjustment in the multivariable analysis.

**Supplementary Table 5**. Uni- and multivariable regression analyses for PFS (patients treated with trastuzumab were excluded) according to residual ctDNA at follow up after 9 weeks of treatment.

|  | N | **Univariable analysis** | | | **Multivariable analysis** | | |
| --- | --- | --- | --- | --- | --- | --- | --- |
|  |  | HR | 95% CI | P | HR | 95% CI | P |
| **Residual ctDNA detectable at follow up**  <1%  ≥1% | 20  7 | **Ref**  **1.87** | **0.76-4.59** | **0.171** | 4.08 | 1.31-12.75 | 0.016 |
| **Age at diagnosis** | - | **0.93** | **0.88-0.97** | **0.001** | 0.89 | 0.84-0.95 | <0.001 |
| **WHO performance score**  WHO PS 0  WHO PS 1  WHO PS 2 | 20  6  1 | *Ref.*  1.53  4.34 | 0.60-3.94  0.51-36.91 | 0.378  0.179 |  |  |  |
| **Albumin** | - | 0.89 | 0.75-1.06 | 0.203 |  |  |  |
| **LDH** | - | 1.00 | 1.00-1.01 | 0.280 |  |  |  |
| **Primary tumor site**  Esophagus  Stomach  GE Junction | 22  1  4 | **Ref.**  **0.31**  **0.31** | **0.04-2.41**  **0.10-0.96** | **0.261**  **0.042** | 0.061  0.206 | 0.01-0.72  0.06-0.74 | 0.027  0.015 |
| **Number of metastatic** **sites**  1  2  ≥3 | 10  10  7 | Ref.  0.93  0.83 | 0.37-2.33  0.31-2.21 | 0.872  0.706 |  |  |  |
| **First-line treatment**  CapOx  CapOx-nabPaclitaxel | 12  15 | Ref.  1.29 | 0.56-3.00 | 0.551 |  |  |  |
| **Previous surgery**  No  Yes | 21  6 | Ref.  1.60 | 0.62-4.12 | 0.33 |  |  |  |
| **Previous chemo(radio)therapy**  No  Yes | 13  14 | Ref.  1.73 | 0.80-3.78 | 0.166 |  |  |  |

Variables in bold are used for adjustment in the multivariable analysis.
